# The recent and future health burden of the U.S. mobile sector apportioned by source

**DOI:** 10.1088/1748-9326/ab83a8

**Published:** 2020-07-06

**Authors:** Kenneth Davidson, Neal Fann, Margaret Zawacki, Charles Fulcher, Kirk R. Baker

**Affiliations:** 1 US EPA, Office of Transportation and Air Quality, Air 4-1 94105, San Francisco, CA, United States of America; 2 US EPA, Office of Air Quality Planning and Standards, Research Triangle Park, NC 27711, United States of America; 3 US EPA, Office of Transportation and Air Quality, Ann Arbor, MI 48105, United States of America

**Keywords:** transportation, particulate matter, ozone, health burden, air quality, mobile sources

## Abstract

Mobile sources emit particulate matter as well as precursors to particulate matter (PM_2.5_) and ground-level ozone, pollutants known to adversely impact human health. This study uses source-apportionment photochemical air quality modeling to estimate the health burden (expressed as incidence) of an array of PM_2.5_- and ozone-related adverse health impacts, including premature death, attributable to 17 mobile source sectors in the US in 2011 and 2025. Mobile sector-attributable air pollution contributes a substantial fraction of the overall pollution-related mortality burden in the U.S., accounting for about 20% of the PM_2.5_ and ozone-attributable deaths in 2011 (between 21 000 and 55 000 deaths, depending on the study used to derive the effect estimate). This value falls to about 13% (between 13 000 and 37 000 deaths) by 2025 due to regulatory and voluntary programs reducing emissions from mobile sources. Similar trends across all morbidity health impacts can also be observed. Emissions from on-road sources are the largest contributor to premature deaths; this is true for both 2011 (between 12 000 and 31 000 deaths) and 2025 (between 6700 and 18 000 deaths). Non-road construction engines, C3 marine engines and emissions from rail also contribute to large portions of premature deaths. Across the 17 mobile sectors modeled, the PM_2.5_-attributable mortality and morbidity burden falls between 2011 and 2025 for 12 sectors and increases for 5. Ozone-attributable mortality and morbidity burden increases between 2011 and 2025 for 10 sectors and falls for 7. These results extend the literature beyond generally aggregated mobile sector health burden toward a representation of highly-resolved source characterization of both current and future health burden. The quantified future mobile source health burden is a novel feature of this analysis and could prove useful for decisionmakers and affected stakeholders.

## 1. Introduction

The risks to human health from exposure to ground-level ozone (O_3_)and fine particles (sized 2.5 microns or smaller, or PM_2.5_) is well established in a large and growing body of literature [1, 2]. Risk assessments, which characterize the number and distribution of air pollution effects among the population, estimate a substantial number of early deaths, hospital admissions, emergency department visits, cases of aggravated asthma and other effects associated with population exposure to these pollutants [3–5]. These assessments commonly report tens of thousands of early deaths and thousands to hundreds of thousands of morbidity effects.

A subset of these assessments have applied photochemical source apportionment modeling techniques to simulate the O_3_ and PM_2.5_ concentrations attributableconcentratio to various sectors and then quantified the risks associated with these concentrations [5, 6]. For example, studies by Fann *et al* and by Caiazzo *et al* each used photochemical source apportionment modeling techniques to inform an air pollution risk assessment characterizing the PM_2.5_ and O_3_ health impacts attributable to a variety of industrial point, area and mobile sources in the U.S [5, 7]. Both studies found that mobile sources, including on-road vehicles, non-road vehicles and ships, were among the largest sources of air pollution health burden.

Building upon this work, we leverage a recently published analysis by Zawacki *et al* in which the authors applied photochemical source apportionment modeling techniques to simulate current and future (years 2011 and 2025) O_3_ and PM_2.5_ concentrations attributable to sources within the U.S. transportation sector [8]. Wolfe *et al* pplied these photochemical model simulations to estimate the dollars in health benefits per ton of reduced O_3_ and PM_2.5_ precursor emissions across 16 categories of mobile sources; these included aircraft, marine vessels, lawn and garden, pleasure craft, heavy duty diesel on-road vehicles, trains and other sources [9]. Here we extend this work by characterizing the size and distribution of the mortality and morbidity impacts on human health.

This manuscript aims to answer the following two questions:

- What is the overall burden to human health associated with the mobile sector in the U.S., and how does this burden vary across the sources within this sector?
- How is this burden distributed across locations?

## 2. Materials and methods

### 2.1 Mobile source emissions and photochemical source apportionment modeling

The estimation of health burden related to mobile sector pollutant emissions described in this paper relies on the source apportionment photochemical modeling simulations described in Zawacki *et al*. Zawacki *et al* modeled the contributions to ambient concentrations of PM_2.5_ and ozone by mobile sector for both 2011 and 2025, the details of which we briefly review here [8].

As Zawacki *et al* documented, emission inputs for the modeling analysis were based on a 2011 emissions inventory; 2025 emissions were projected from the 2011 inventory. Non-mobile source emissions were taken from EPA’s 2011 v6.2 emissions modeling platform, which is based on version 2 of the 2011 National Emissions Inventory (NEI) [10].

Mobile source emissions were categorized into 17 sectors based on fuel use, vehicle and engine type, and are presented in table 1. Onroad emissions inventories were generated using the Motor Vehicle Emission Simulator (MOVES2014) [11]. MOVES inputs were based on state submittals to the NEI or generated by the EPA using national defaults. Locomotive emissions for 2011 were developed by applying growth factors to 2008 NEI values based on freight traffic data. Commercial marine vessel inventories for 2011 were developed using a 2002 base year inventory and regional growth factors. Aircraft emissions cover commercial aircraft landing and take-off emissions up to 3000 feet, and aircraft ground support emissions at airports. Aircraft emissions at altitudes above 3000 feet are not included. Emissions for other nonroad engines and equipment, such as lawn and garden equipment, construction equipment, commercial, and agricultural engines, were generated using the NONROAD 2008 model [12].

The only exception with respect to generation of the 2011 inventory is California. California’s onroad emissions were based on the EMFAC2011 model estimates provided by the state of California [13]. Aircraft, rail, and marine inventories for California were provided by the state of California. Emissions for other nonroad engines and equipment were generated using the OFFROAD model for California [14].

Emissions were projected to 2025 using information on growth, activity and fleet turnover; details on these projections can be found in Zawacki *et al* and are available in the technical support document for Preparation of Emissions Inventories for the Version 6.2, 2011 Emissions Modeling Platform [8, 15]. Projections account for emission reductions expected from regulations that were final at the time that the platform was finalized, including the following mobile source regulations: Final Rule for Control of Air Pollution from Motor Vehicles: Tier 3 Motor Vehicle Emission and Fuel Standards (79 FR 23414, 28 April 2014), New Marine Compression-Ignition Engines at or Above 30 l per Cylinder Rule (75 FR 22895, 30 April 2010), the Marine Spark-Ignition and Small Spark-Ignition Engine Rule (73 FR 59034, 8 October 2008), the Locomotive and Marine Rule (73 FR 25098, 6 May 2008), the Clean Air Nonroad Diesel Rule (69 FR 38957, 29 June 2004), the Heavy-Duty Engine and Vehicle Standards and Highway Diesel Fuel Sulfur Control Requirements (66 FR 5002, 18 January 2001) and the Tier 2 Motor Vehicle Emissions Standards and Gasoline Sulfur Control Requirements (65 FR 6698, 10 February 2000). Projections also include reduced emissions that result from existing local inspection and maintenance (I/M) and other onroad mobile programs, such as California LEVIII, the National Low Emissions Vehicle (LEV) and Ozone Transport Commission (OTC) LEV regulations, local fuel programs, and Stage II refueling control programs. The projections also account for stationary source regulations that were final at the time the platform was finalized; for example, the projected electric generating unit (EGU) emissions include the Final Mercury and Air Toxics (MATS) rule and the Cross-State Air Pollution Rule (CSAPR) but not the Clean Power Plan.

Using these emissions as inputs, Zawacki and co-authors ran the Comprehensive Air Quality Model with extensions (CAMx) v6.2 for their analysis. Zawacki *et al* simulated the O_3_ and PM_2.5_ concentrations from 20 sectors in total, 17 of which were mobile source sectors (as described in table 1). The three non-mobile sectors include: biogenics, fugitive dust and agricultural ammonia; ”all other sectors”; and initial/boundary conditions.

Detailed information regarding the evaluation of the performance of the CAMx model may be found in Zawacki *et al*, as well as maps illustrating the O_3_ and PM_2.5_ concentrations for each mobile source sector [7].

Following EPA-established methods, we use the CAMx modeling simulations in a relative sense by ‘anchoring’ predicted base-year concentrations (2011) to observed ambient values collected at monitoring locations and then estimate relative reduction factors (RRFs) using the future (2025) simulations to project future concentrations [16, 17]. This approach addresses potential model bias and error in the base year simulations and assumes that factors causing bias (either under- or over-predictions) in the base case also affect the future case. Monitor-level concentrations are interpolated to the 12 km by 12 km model grid for the contiguous 48 states and multiplied by the RRFs to generate a future year air quality surface. These 2011 and 2025 PM_2.5_ and ozone concentrations are used as inputs to the assessment of health burden associated with each mobile source sector.

### 2.2. Health impact assessment

We follow a well-established technique for quantifying O_3_ and PM_2.5_-related effects by using health impact functions [8, 18]. A health impact function calculates the excess number of air pollution-related premature deaths or illnesses using four terms: (1) a beta coefficient, derived from a relative risk, odds ratio or Hazard Ratio from a published air pollution epidemiologic study; (2) an estimated pollutant concentration; (3), a count of individuals exposed to that pollutant; and (4) the baseline rate of death or disease among that population matched to the health endpoint of interest. We calculated the health impact function using the Benefits Mapping and Analysis Program—Community Edition (BenMAP-CE, v1.3) tool as described below [19, 20].

Below we illustrate the steps to calculating the health impact function using PM_2.5_-related premature death as an example (equation (1)). Here we estimated the number of PM_2.5_-related total deaths (*y*_*ij*_) in each year i (*i* = 2011, 2025) among adults aged 30 and above in each county j (*j* = 1,…,J where *J* is the total number of counties) as

(1) $${\text{y}}_{\text{ij}}=\sum {\text{ay}}_{\text{ija}}\;{\text{y}}_{\text{ija}}={\text{m}}_{\text{ija}}\times \left(e^{\beta \cdot {\text{C}}_{\text{ij}}-1}\right)\times {\text{P}}_{\text{ija}},$$

where *β* is the risk coefficient for all-cause mortality for adults in association with PM_2.5_ exposure, *m*_*ija*_ is the baseline all-cause mortality rate for adults aged *a* = 30–99 in county j in year i stratified in 10 year age bins, *C*_*ij*_ is annual mean PM_2.5_ concentration in county j in year i, and *P*_*ija*_ is the number of county adult residents aged *a* = 30–99 in county j in year i stratified into 5 year age bins.

We defined *m*_*ija*_ as the county-level age-stratified all-cause death rates from the Centers for Disease Control Wide-ranging Online Data for Epidemiologic Research database [21]. To account for the improving longevity of the population, we projected these death rates to future years using a life table reported by the U.S. Census Bureau; more details regarding procedure can be found in Fann *et al* [22, 23]. We defined the baseline incidence rates for the morbidity endpoints using rates of hospital admissions, emergency department visits and other outcomes for the year 2014.

We defined *P*_*ija*_ using age-, sex- and race-stratified population data from the U.S. Census Bureau. To account for growth in the size of the U.S. population, we used demographic forecasts from the Woods & Poole company to project the U.S. Census population counts from the year 2010 to the year 2011 and 2025 [24].

To characterize uncertainty in the estimated counts of avoided deaths and illnesses, we performed a Monte Carlo simulation, sampling from the standard error reported in the epidemiological study for each beta coefficient; this produced an error distribution of estimated PM_2.5_ and O_3_-related effects. We estimated total numbers of premature deaths and illnesses in the continental U.S. for each year by summing the county-specific estimates and report the sums of the 2.5th and 97.5th percentiles of the Monte Carlo distributions as 95% confidence intervals.

When calculating equation (1) we assume that the association between the pollutant and each health outcome is log-linear over the entire range of PM_2.5_ exposure, with no level below which PM_2.5_ would not increase the risk of death [1, 25]. The lowest measured level included in the long-term exposure study used to quantify PM-related risks is 5.8 *μ*gm^−3^ and thus we extrapolated the portion of the curve below this level [26]. The β coefficient for each O_3_ and PM_2.5_ mortality and morbidity endpoint can be found in the supplemental materials (supplemental tables S1 and S2 (https://stacks.iop.org/ERL/15/075009/mmedia)). Baseline incidence rates for the full suite of O_3_ and PM_2.5_ endpoints can also be found in the supplemental materials (supplemental tables S3 and S5).

We calculated the fraction of all deaths due to PM_2.5_ in each county j and year i using the following function:

(2) $$AF_{ij}=\frac{y_{ij}}{\underset{a}{\sum }mo_{ija}\times P_{ija}},$$

where *y*_*ij*_ is the estimated number of air pollution deaths in county *j* in year *i, m*_*ija*_, is the age-stratified baseline death rate, and, *P*_*ija*_ is the age-stratified population, respectively, in county *j* in year *i*.

## 3. Results

In this analysis, health burden is characterized by counts of premature deaths attributable to directly emitted PM_2.5_ and PM_2.5_ precursor emissions, premature deaths attributable to ozone precursor emissions, and a full suite of PM_2.5_ and ozone-related morbidity (non-fatal) health impacts. In terms of premature mortality (table 2), the mobile sector contributes a substantial fraction of the overall PM_2.5_ and ozone air pollution health burden in the U.S., accounting for about 20% (21 000 deaths) of total PM_2.5_ and ozone-attributable deaths in 2011 (110 000 deaths, of which approximately 90 000 are attributed to non-mobile sources). This value falls to about 13% (13 000 deaths) of the total PM_2.5_ and ozone-attributable deaths by 2025 (110 000 deaths, of which approximately 100 000 are attributed to non-mobile sources) due to regulatory and voluntary programs reducing emissions from mobile sources. These trends demonstrate that over time, the premature mortality health burden associated with reductions in mobile source pollution is decreasing while the premature mortality burden from all non-mobile sources of pollution combined is projected to increase over that same time period. Similar trends across all morbidity health impacts can also be observed. Tables 3 and 4 present the full suite of quantified health burden incidence endpoints (mortality and morbidity) in 2011 and 2025, respectively, aggregated across the non-road, on-road, and air/rail/marine sectors. Sector-specific results can be found in supplemental tables S6 and S7.

**Figure 1.**
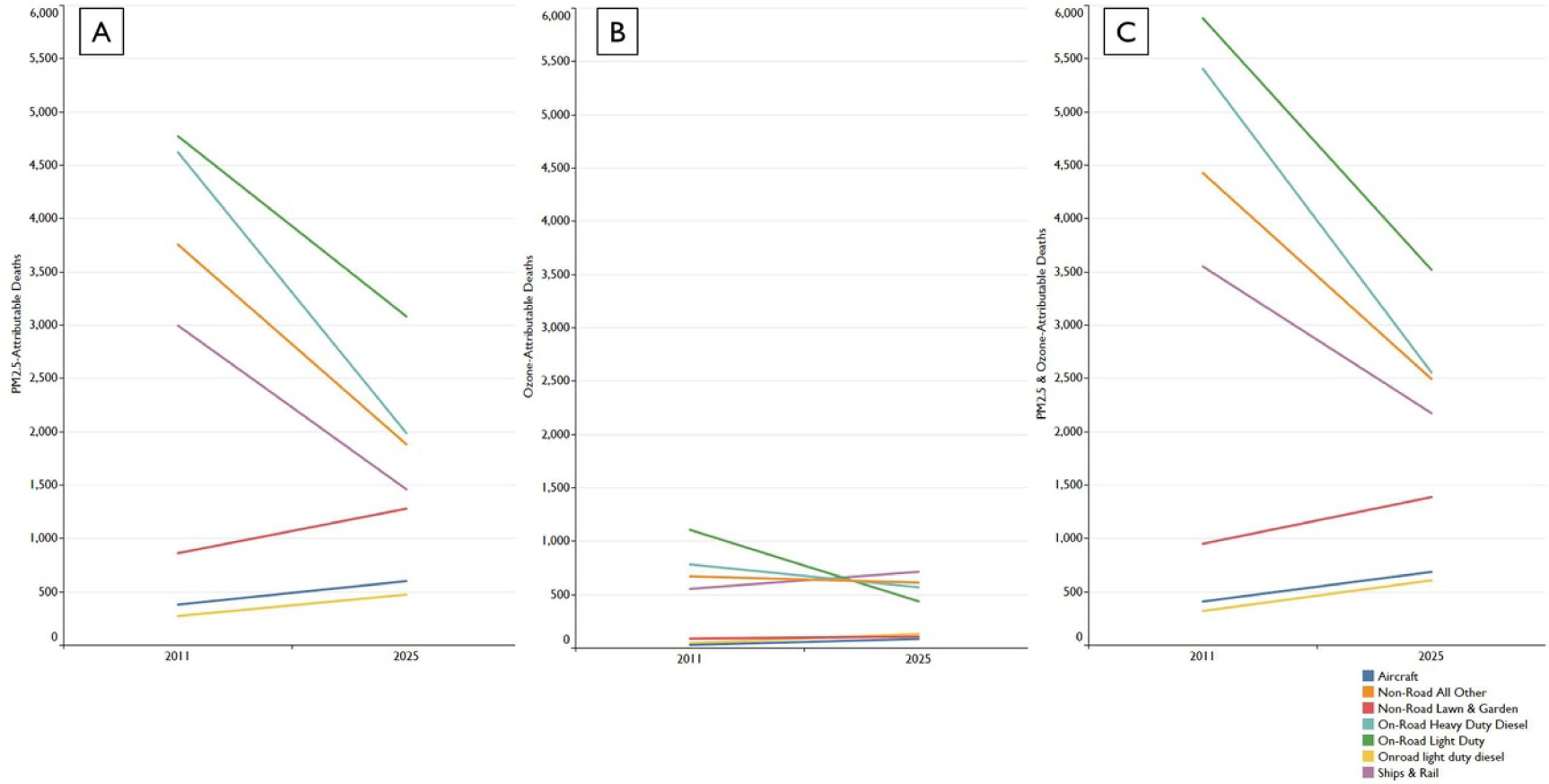
Trend of air pollution-related deaths over time by pollutant and mobile sector source. Each colored line represents a subset of 17 separate mobile emissions sources. Panel A presents PM_2.5_-related deaths over time by sector. Panel B presents ozone-related deaths over time by sector. Panel C presents total (PM_2.5_ + ozone) deaths over time by sector.

Among the mobile sources modeled, the total premature mortality burden from on-road sources (light-duty gas cars and motorcycles; light duty gas trucks; heavy duty gas trucks; light duty diesel trucks; and, heavy duty diesel trucks) is the greatest (table 2; figure 1); this is true for both 2011 and 2025. Non-road construction engines, C3 marine engines and emissions from rail also contribute to large portions of mobile source mortality burden. Across the 17 mobile sectors modeled, the PM_2.5_-attributable mortality burden falls between 2011 and 2025 for 12 sectors and increases for 5. The C3 marine, nonroad construction and nonroad agricultural sectors experience the greatest reduction in PM-related burden over this time period, while light duty diesel and aircraft (including ground support) experience the greatest growth.

**Figure 2.**
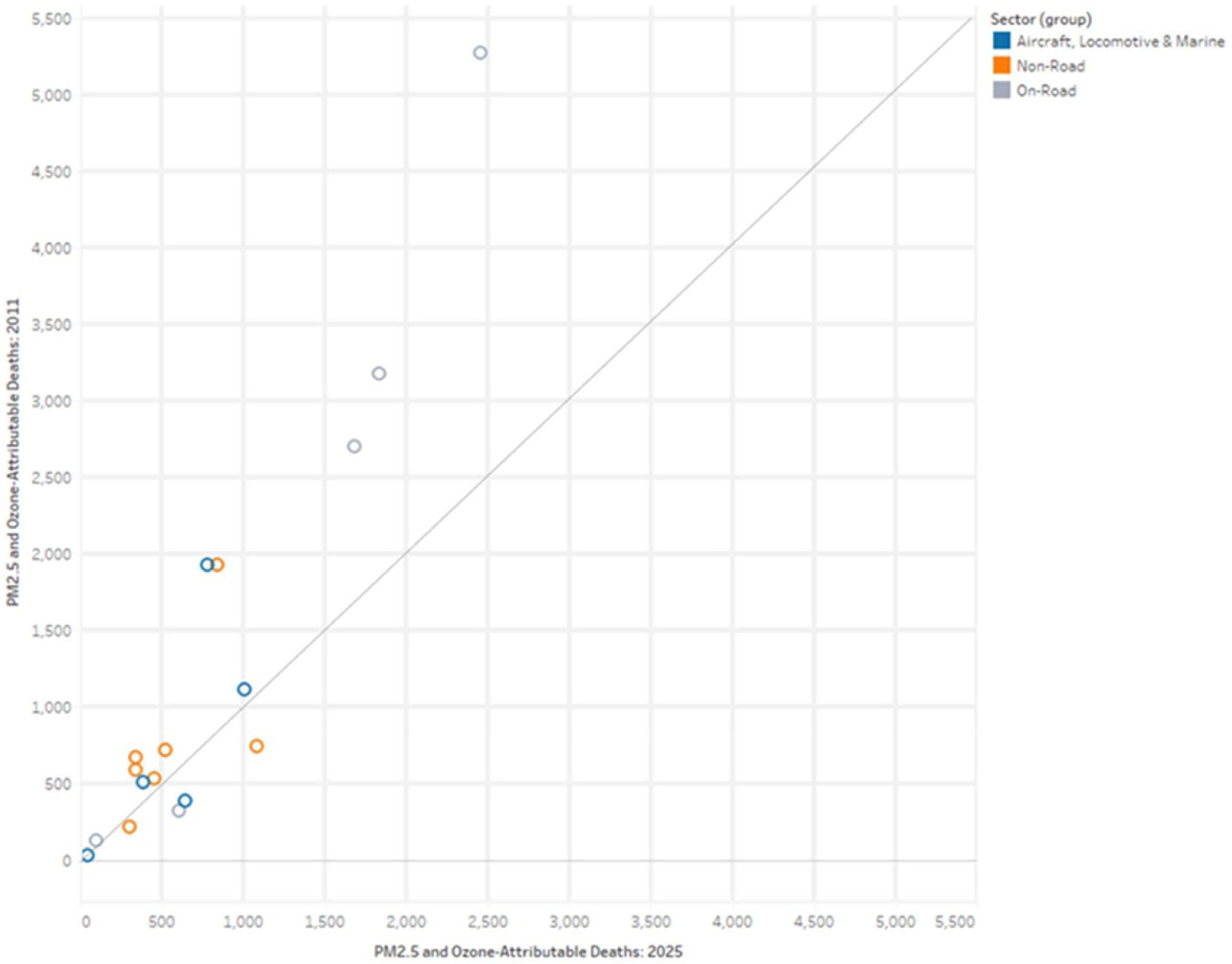
Mobile source-related deaths (PM_2.5_ + ozone) by sector in 2011 compared to 2025.

Compared to PM_2.5_-attributable mortality burden from mobile sources, the burden associated with deaths from ozone is much smaller—contributing to approximately 16% of total mobile source-related mortality burden in 2011 and 20% in 2025. Across the 17 mobile sectors, ozone-attributable mortality burden increases between 2011 and 2025 for 10 sectors and falls for seven (table 2, figure 1). Total mortality burden (PM_2.5_ + ozone) falls between 2011 and 2025 for 12 mobile source sectors and increases for 5 (table 2, figure 2).

**Figure 3.**
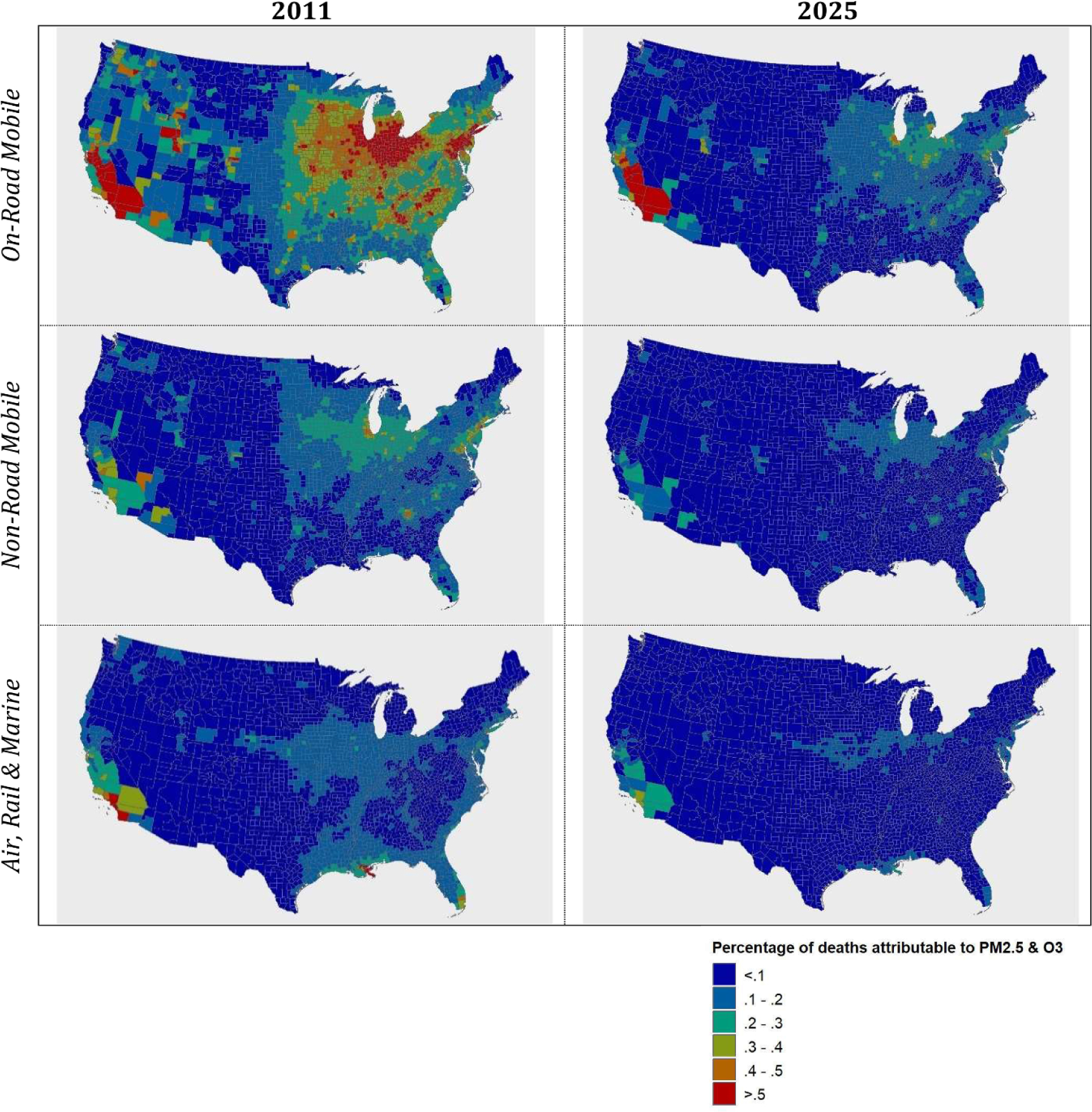
Percentage of deaths due to PM_2.5_ and ozone from the on-road, non-road and aircraft, locomotive and marine mobile sectors in 2011 and 2025.

To illustrate the spatial distribution of mobile sector public health burden, we provide maps of the fraction of all deaths attributable to mobile source PM_2.5_ and ozone-related mortality impacts. The fraction of pollution-related deaths attributable to the on-road mobile, non-road mobile and the air, locomotive and marine sectors is as large as about 0.5% (figure 3). The fraction of deaths attributable to the on-road mobile sector is the most substantial—in terms of both the magnitude of risk and geographic scope of the impact. For 2011, we find that large portions of California, the Midwest and Northeast experience among the greatest fraction of deaths due to on-road related PM_2.5_ and ozone, as compared both to other parts of the U.S and to the other mobile sectors (figure 3). These results are consistent with characteristics associated with the on-road sector; on-road vehicle traffic and roadway density are most pronounced in areas with the largest population density.

The attributable fraction for the non-road mobile sector has a similar geographic pattern, tracking with population density, though the size of the attributable fraction is significantly smaller than it is for the on-road sector. Finally, the attributable fraction for the air, locomotive and marine sector tends to affect discrete portions of the U.S., most significantly near Los Angeles, New Orleans and Miami. Rail and C1C2 marine impacts can also be discerned, tracking with rail lines and coastal, river and lake locations, especially in 2011.

In any given location, the magnitude of premature deaths will be influenced by the combination of air quality, population density, and baseline health status. On a per-person basis, this will vary by mobile source sector depending on engine use characteristics. Generally, southern California, the industrial Midwest, and the urban Northeast corridor see the greatest exposures to mobile source pollutants and therefore the largest fraction of mobile source pollution-related deaths (figure 3). We note that the estimated ozone-related mortality impacts are an order of magnitude smaller than those estimated for PM_2.5_, partly due to the smaller relative risk associated with ozone, and partly due to the fact that ozone is more geographically dispersed as a completely secondarily-formed pollutant.

## 4. Discussion

Emissions trends explain the overall sector-specific health burden trends. For example, phase-in of promulgated regulations and vehicle fleet turnover, particularly in the on-road sector, lead to net reductions in total future emissions. An exception is the light-duty diesel sector, which is projected to have slightly higher future emissions due to growth in population and vehicle miles traveled. Growth in air travel and airport-related activity without recent regulatory control of emissions explain the increase in burden for these sectors. Fewer regulatory controls on non-road emissions help explain why this sector contributes to a larger fraction of total mobile source emissions in the future (e.g. no recent non-road diesel engine or lawn and garden equipment regulations), as well as urban population growth and land-use changes.

Spatial trends follow closely with engine use and vehicle type. As described above, the on-road mobile sector is responsible for the largest percentage of mobile source health burden and tracks closely with the U.S. highway network. The density of traffic in highly urbanized areas, as well as interstate travel and the ubiquity of on-road vehicle emissions help explain the peaks in urban health burden and the large spatial gradient of burden that extends across much of the continental U.S.

Past source apportionment work is useful in terms of providing insight into the relative contribution emissions from different source sectors contribute to health risk. However, the risk from mobile sources are often aggregated into broad categories encompassing different types of vehicles and engine technology. For instance, in Fann *et al* ‘mobile sources’ included all vehicle types, including on-road and non-road vehicles, as well as aircraft, rail and marine sources [7]. Such a broad category does not provide the information one would need to examine how different mobile source sectors contribute to health burden and how this contribution to burden changes over time and space.

Fann *et al* estimated that in 2016, the total (PM_2.5_ + ozone) burden of premature death attributable to the mobile sector ranged between 19 000 and 54 000, which is comparable to total mobile source mortality burden measured in this paper: between 21 000–55 000 in 2011 and 13 000–37 000 in 2025, depending on the source of the effect estimate derived from the literature [7]. The comparability of these estimates is not surprising; both analyses used similar emissions sources, air quality models, source apportionment approach, and similar health impact assessment methods and inputs, though input data for this analysis was updated when appropriate and where more recent data was available.

Another study, which used different emissions inventories, meteorology, air quality models, apportionment approach, and health assessment assumptions, estimated the burden of premature death (PM_2.5_ + ozone) from seven broad source sectors in 2005, including ‘road’ (58 000), ‘marine’ (8800), ‘rail’ (5000) and ‘aviation’ (1400) [5]. These estimates are higher than the attributable burden estimated in this paper, though emissions for several mobile source categories were likely larger in 2005 due to the absence of more recent regulations and the ongoing phase-in of promulgated mobile source emission regulations.

A recent analysis estimated PM_2.5_-related mortality burden for many disaggregated emission sources as a metric used to quantify racial-ethnic disparities in the generation of emissions and resultant exposure to those emissions [31]. Though different air quality modeling tools, methods and input data were used, Tessum et al’s estimate of mobile source-related premature deaths in 2015 (approximately 35 000) fall within the range of mobile sector deaths estimated in this analysis.

In this analysis, we estimate the total burden of premature death associated with all sources of PM_2.5_-related emissions to range between 100 000 and 220 000 in 2011 and 99 000 and 220 000 in 2025 (the similarity in incidence is coincidental, related to offsetting emissions changes over time across source categories). ‘All sources’ is defined as the sum of burden across the 17 mobile source categories plus the additional ‘All non-mobile’ source categories defined in table 1. This estimated health burden is comparable to recent estimates in the published literature. For example, Goodkind *et al* estimated that anthropogenic PM_2.5_ was responsible for 107 000 deaths in 2011 [32]. Tessum *et al* estimated that 102 000 premature deaths were associated with anthropogenic PM_2.5_ in 2015 [31]. Fann *et al* estimated that in 2005, ambient PM_2.5_ was associated with approximately 120 000 premature deaths. The Global Burden of Disease study (Burnett *et al* 2018) estimates that in 2017, ambient PM_2.5_ pollution is related to approximately 84 000 premature deaths [33]. Dedoussi *et al* estimated U.S. combustion emission-related premature deaths across seven broad sectors, finding 83 300 attributable deaths in 2011 and 66 100 in 2018 [34]. This general consistency with prior estimates gives us greater confidence in the magnitude of the effects estimated here and suggests that recent and future levels of ozone and PM_2.5_ still pose a public health risk in many regions of the United States.

Uncertainties and limitations exist at each stage of the emissions-to-health burden analysis (e.g. emissions inventory uncertainty, air quality modeling uncertainty, health impact assessment uncertainty). The air quality modeling relied on emissions data generated using MOVES2014, an older version of EPA’s current mobile source emissions tool—MOVES2014b [11]. MOVES2014b includes non-road sector updates that result in lower national-level criteria pollutant emissions. We would therefore expect the portion of burden attributed to nonroad sources to be reduced by a small percent if the analysis was updated to include nonroad emissions estimated using MOVES2014b. More generally, inventories for some emission sources, including rail and marine, are less certain than inventories for onroad sources, resulting in uncertainties in health burden estimates across source sectors. Detailed air quality model evaluation is performed in [8].

A number of uncertainties associated with the assessment of criteria pollutant-related health impacts are systematic across sectors and pollutants, including those associated with assumptions about the causal relationship between PM_2.5_ exposure and premature mortality (especially at lower concentrations) and the shape of the chosen concentration response functions. Other sources of uncertainty may have heterogeneous impacts across pollutant sources and species, such as the variation in effect estimates reflecting differential toxicity of particle components and regional differences in pollutant composition, though there currently is insufficient scientific evidence to differentiate health effect estimates by emission species [35]. Health impact assessments are an integral part of regulatory impact assessments and provide a useful reference about the contributors to and magnitude of uncertainty present in health burden estimates [36, 37].

Despite these uncertainties, the estimates of attributable health burden presented here provide reasonable estimates of the magnitude of adverse health impacts associated with recent and future emissions from a broad class of mobile sources. Compared to values reported elsewhere, these estimates of health impacts extend the literature beyond generally aggregated mobile sector health burden toward a representation of highly-resolved source characterization of both current and future health burden conditions. The ability to predict future mobile source health burden that reflects modeled trends in sector-specific emissions is a novel feature of this analysis and could prove useful for decisionmakers and affected stakeholders when considering how to address and prioritize emission controls across the mobile source sector.

## Supplementary Material

Published Supplementary Information

## Figures and Tables

**Table 1.** Projected 2011 and 2025 emissions (short tons) from mobile sectors tracked for contribution.

	VOC		NO_X_		CO		SO_2_		Primary PM_2.5_		NH_3_	
Description	2011	2025	2011	2025	2011	2025	2011	2025	2011	2025	2011	2025
Nonroad recreational (incl. pleasure craft)	1 273 517	584 652	168 439	163 443	3 815 598	3 187 882	549	423	29 762	13 051	386	422
Nonroad construction	77 690	52 066	531 560	189 821	652 604	427 675	1241	461	44 580	12 708	548	712
Nonroad lawn & garden commercial	249 164	248 135	67 648	54 215	3 352 063	3 729 786	279	136	16 025	19 164	200	255
Nonroad lawn & garden residential	205 867	153 771	26 996	19 777	2 305 837	2 534 336	140	86	4380	5675	107	145
Nonroad agriculture	56 812	27 377	482 559	191 440	450 552	243 007	900	340	38 579	11 310	476	581
Nonroad commercial	136 417	98 805	119 561	74 653	2 484 411	2 790 930	316	179	9114	6173	364	554
Nonroad all other: (industrial, logging, mining, oil field)	49 951	23 816	221 232	98 772	928 098	414 700	582	457	11 324	4348	532	852
*Nonroad Subtotal*	*2 049 418*	*1 188 622*	*1 617 995*	*792 121*	*13 989 163*	*13 328 316*	*4007*	*2082*	*153 764*	*72 429*	*2613*	*3521*
Onroad light duty gas cars and motorcycles	1 025 647	381 387	1 066 945	219 726	9 324 076	3 999 261	10 949	3487	31 348	19 814	56 042	34 090
Onroad light duty gas trucks	1 372 896	398 441	1 893 768	337 035	14 996 186	5 150 855	14 811	4665	35 479	22 274	57 349	38 298
Onroad heavy duty gas + compressed natural gas (CNG)	47 439	21 056	90 250	30 095	852 807	447 225	471	197	1512	1164	914	1049
Onroad Light duty diesel	42 470	40 564	127 106	173 650	376 015	478 816	272	1291	6581	6692	769	4789
Onroad Heavy duty diesel	221 018	97 316	2 542 140	946 522	764 741	371 279	3163	3748	119 770	30 201	5731	7224
*Onroad Subtotal*	*2 709 470*	*938 764*	*5 720 209*	*1 707 028*	*26 313 825*	*10 447 436*	*29 666*	*13 388*	*194 690*	*80 145*	*120 805*	*85 450*
Category 1 and 2 (c1c2) marine	13 812	8310	561 321	305 350	114 623	112 451	8103	795	18 012	9069	348	351
Category 3 (c3) marine	32 960	58 707	870 291	833 822	77 992	138 679	346 493	57 096	39 624	12 063	68	68
Rail	46 247	23 317	866 717	582 351	136 220	171 163	8528	382	26 200	13 445	376	375
Aircraft (excluding ground support)	27 360	32 918	104 766	140 528	402 463	454 691	12 453	16 627	7281	8074	0	0
Aircraft ground support only	2283	3152	7606	10 492	66 577	91 976	201	279	233	321	0	0
*Aircraft/Rail/Marine Subtotal*	*122 662*	*126 404*	*2 410 701*	*1 872 543*	*797 875*	*968 960*	*375 778*	*75 179*	*91 350*	*42 972*	*792*	*794*
Mobile sector total	4 881 548	2 253 790	9 748 904	4 371 692	41 100 866	24 744 712	409 450	90 648	439 803	195 548	124 210	89 765
All non-mobile sectors	52 805 510	57 737 923	6 435 179	6 368 880	35 914 792	33 416 351	6 257 818	3 249 966	4 197 142	4 230 808	4 063 804	4 176 621
*Mobile total as percent of total emission inventory*	8%	4%	64%	41%	53%	43%	6%	3%	10%	4%	3%	2%

Notes: Category 1 and 2 marine engines are vessels with engines with a cylinder displacement between 2.5 and 30 l.

Category 3 marine engines are large ocean-going vessels with engines with a cylinder displacement greater than 30 l.

‘All non-mobile sectors’ aggregates emissions from three non-mobile source categories: (1) biogenics, fugitive dusts, and agricultural ammonia; (2) ‘all other sectors’, which includes emissions from oil and gas exploration, non-Electricity Generating Unit point, Electricity Generating Unit point, non-point, fires (wild, prescribed, agricultural), and biomass burning; and (3) initial/boundary condition emissions.

**Table 2.** Estimated number of PM_2.5_ and ozone-related premature deaths attributable to each of 17 mobile sectors in 2011 and 2025 (95% confidence intervals)^a^

	2011		2025		Change in Burden^b^		
	PM_2.5_	Ozone	PM_2.5_	Ozone	PM_2.5_	Ozone	Total
***Non-road***							
Recreational (incl. pleasure craft)	520 (350–690)	200 (70–350)	310 (210–410)	210 (70–350)	↓41%	↑8%	↓28%
Construction	1800 (2300–680)	170 (55–280)	700 (470–920)	140 (46–230)	↓60%	↓17%	↓56%
Lawn & garden commercial	680 (460–900)	56 (19–94)	1000 (680–1300)	78 (26–130)	↑48%	↑38%	↑47%
Lawn & garden residential	180 (120–240)	32 (11–53)	270 (180–360)	30 (10–50)	↑50%	↓6%	↑41%
Agriculture	480 (320–630)	190 (63–320)	210 (140–270)	130 (44–220)	↓57%	↓29%	↓49%
Commercial	490 (330–650)	46 (15–76)	400 (270–530)	57 (19–96)	↓19%	↑26%	↓15%
All other (industrial, logging, mining, oil)	510 (350–680)	74 (25–120)	270 (180–360)	71 (23–120)	↓47%	↓5%	↓42%
***On-road***							
Light duty gas cars and motorcycles	2300 (1600–3000)	410 (140–680)	1500 (1000–2000)	180 (58–290)	↓34%	↓57%	↓38%
Light duty gas trucks	2500 (1700–3300)	700 (230–1200)	1600 (1100–2100)	260 (87–440)	↓37%	↓62%	↓42%
Heavy duty gas + CNG	96 (65–130)	32 (11–53)	79 (53–100)	23 (8–38)	↓18%	↓29%	↓21%
Light duty diesel	270 (190–360)	47 (16–78)	480 (320–630)	130 (44–220)	↑73%	↑182%	↑89%
Heavy duty diesel	4500 (3100–6000)	750 (250–1300)	1900 (1300–2500)	550 (180–910)	↓58%	↓27%	↓54%
***Marine, rail and aircraft***							
C1 & C2	380 (260–500)	130 (43–220)	260 (170–340)	130 (44–220)	↓32%	↑1%	↓23%
C3	1700 (1200–2300)	220 (74–370)	460 (310–600)	320 (110–540)	↓73%	↑44%	↓60%
Rail	910 (620–1200)	200 (67–340)	750 (500–990)	260 (86–430)	↓18%	↑29%	↓10%
Aircraft (excluding ground support)	360 (240–470)	26 (9–43)	570 (380–750)	78 (26–130)	↑58%	↑200%	↑68%
Aircraft ground support only	23 (15–30)	3 (1–5)	33 (24–44)	8 (3–14)	↑49%	↑173%	↑64%
***All non-mobile sector***^c^	83 000 (56 000–110 000)	8800 (2900–15 000)	88 000 (60 000–120 000)	12 000 (3900–20 000)	↑6%	↑34%	↑9%

a Incidence estimates rounded to two significant figures. PM-related premature deaths based on effect estimates derived from Krewski *et al* 2009 26]; ozone-related premature deaths based on effect estimates derived from Bell *et al* 2004 [27]. Attributable deaths estimated using population projected to each year.

b Percent change calculated on estimates prior to rounding.

c ‘All Non-mobile Sectors’ includes biogenics, fugitive dusts, agricultural ammonia, oil and gas exploration, non-Electricity Generating Unit point, Electricity Generating Unit point, non-point, fires (wild, prescribed, agricultural), biomass burning, and initial/boundary condition emissions.

**Table 3.** 2011 health burden for aggregated non-road, on-road and air/rail/marine sectors.

	Non-road	On-road	Air/Rail/Marine
*Premature death*			
Krewski *et al* (2009) [26] & Bell *et al* (2004) [27]	5400 (3400–7400)	12 000 (7200–16 000)	4000 (2500–5400)
Lepeule *et al* (2012) [28] & Levy *et al* (2005) [29]	14 000 (7600–20 000)	31 000 (17 000–45 000)	10 000 (5700–15 000)
*Non-fatal heart attacks*			
Peters *etal* (2001) [30]	10 000 (5200–16 000)	22 000 (11 000–33 000)	7700 (3800–11 000)
Pooled estimate of four studies	460 (170–1200)	970 (360–2600)	330 (120–890)
Cardiovascular hospital admissions	1100 (490–1900)	2300 (1000–4100)	790 (350–1400)
Respiratory hospital admissions	2100 ((530)–4400)	5000 ((1300)–10 000)	1600 ((410)–3300)
Respiratory emergency department visits	9200 ((520)–24 000)	21 000 ((710)–62 000)	6700 ((320)–18 000)
Acute bronchitis	7100 ((1700)–16 000)	15 000 ((3600)–34 000)	5200 ((1200)–12 000)
Acute respiratory symptoms	8 700 000 (5 100 000–12 000 000)	20 000 000 (12 000 000–29 000 000)	6 500 000 (3 800 000–9 200 000)
Aggravated asthma	2 300 000 ((1 800 000)–5 500 000)	5 700 000 ((4 700 000)–14 000 000)	1 700 000 ((1 400 000)–4 200 000)
Upper respiratory symptoms	130 000 (24 000–240 000)	280 000 (50 000–500 000)	95 000 (17 000–170 000)
Lower respiratory symptoms	91 000 (35 000–150 000)	190 000 (74 000–310 000)	67 000 (25 000–110 000)
Lost work days	650 000 (550 000–750 000)	1 400 000 (1 200 000–1 600 000)	480 000 (410 000–550 000)
Lost school days	1 600 000 (550 000–3 500 000)	4 000 000 (1 400 000–8 800 000)	1 200 000 (420 000–2 600 000)

**Table 4.** 2025 health burden for aggregated non-road, on-road and air/rail/marine sectors.

	Non-road	On-road	Air/Rail/Marine
*Premature death*			
Krewski *et al* (2009) & Bell *et al* (2004)	3900 (2400–5400)	6700 (4100–9200)	2900 (1700–4100)
Lepeule *et al* (2012) & Levy *et al* (2005)	11 000 (6000–15 000)	18 000 (10 000–26 000)	8500 (5000–12 000)
*Non-Fatal heart attacks*			
Peters *et al* (2001)	3200 (780–5600)	5700 (1400–10 000)	2100 (520–3700)
Pooled estimate of four studies	350 (130–930)	620 (230–1700)	230 (84–610)
Cardiovascular hospital admissions	820 (360–1500)	1500 (640–2700)	540 (240–990)
Respiratory hospital admissions	2100 ((590)–4500)	3600 ((1000)–7500)	2000 ((550)—4400)
Respiratory emergency Department visits	7400 ((180)–20 000)	12 000 ((380)–33 000)	7200 (130–21 000)
Acute bronchitis	4400 ((1000)–9800)	8000 ((1900)–18 000)	2900 ((680)—6500)
Acute respiratory symptoms	6 400 000 (3 600 000–9 300 000)	11 000 000 (6 100 000–15 000 000)	6 100 000 (3 200 000–9 100 000)
Aggravated asthma	1 900 000 ((1 500 000)–4 500 000)	3 000 000 ((2 400 000)–7 200 000)	2 000 000 ((1 700 000)—4 900 000)
Upper respiratory symptoms	80 000 (14 000–140 000)	140 000 (26 000–260 000)	53 000 (9500–95 000)
Lower respiratory symptoms	56 000 (21 000–91 000)	100 000 (39 000–160 000)	37 000 (14 000–60 000)
Lost work days	410 000 (340 000–470 000)	730 000 (620 000–840 000)	270 000 (230 000–310 000)
Lost school days	1 300 000 (460 000–2 900 000)	2 100 000 (740 000–4 600 000)	1 400 000 (510 000–3 200 000)
