# Supplementary material for: The recent and future health burden of the U.S. mobile sector apportioned by source
Source: Environ Res Lett. Author manuscript; Available in PMC 2021 Apr 15. (PMC8048113; doi:10.1088/1748-9326/ab83a8)
Supplement: Published Supplementary Information [file NIHMS1682500-supplement-Published_Supplementary_Information.pdf]

Supplemental Information

Title: The Recent and Future Health Burden of the U.S. Mobile Sector Apportioned by Source

Authors: Kenneth Davidson (corresponding author)[a], Neal Fann[b], Margaret Zawacki[c], Charles Fulcher[b], Kirk R. Baker[b]

[a] US EPA, Office of Transportation and Air Quality, Air 4-1, San Francisco, CA 94105 (davidson.ken@epa.gov; 1-415-972-3633)
[b] US EPA, Office of Air Quality Planning and Standards, Research Triangle Park, NC 27711 (fann.neal@epa.gov, baker.kirk@epa.gov, fulcher.charles@epa.gov)
[c] US EPA, Office of Transportation and Air Quality, Ann Arbor, MI 48105 (zawacki.margaret@epa.gov)

Disclaimer: The views expressed in this article are those of the authors and do not necessarily represent the views or policies of the U.S. Environmental Protection Agency.

Table S-1: Epidemiological Study Parameters Used to Quantify $PM_{2.5}$-Attributable Risks

Endpoint	Study	Study Population	Risk Estimate (95 Percent Confidence Interval)[A]
**Premature Mortality**			
Cohort study, all-cause	Krewski et al. (2009)[1]	>29 years	RR = 1.06 (1.04 – 1.06) per 10 μg/m^3
	Lepeule et al. (2012)[2]	>24 years	RR = 1.14 (1.07 – 1.22) per 10 μg/m^3
**Chronic Illness**			
Nonfatal heart attacks	Peters et al. (2001)[3]	>18 years	OR = 1.62 (1.13 – 2.34) per 20 μg/m^3
**Hospital & Emergency Department Admissions**			
Respiratory	Babin et al. (2007)[4]—ICD 493 (asthma)	<18	β=0.002 (0.004337)
	Moolgavkar (2000)—ICD 490–496 (COPD)[6]	18–64 years	1.02 (1.01—1.03) per 36 μg/m^3
	Zanobetti and Schwartz (2006)[7]—ICD 470-519 (All Respiratory)	>64 years	β=0.00207 (0.00446)
Cardiovascular	*Pooled using equal weights:* Zanobetti and Schwartz (2009)[8]—ICD 390-459 (all cardiovascular)	>64 years	β=0.00189 (0.000283)
	Peng et al. (2009)[9]—ICD 426-427; 428; 430-438; 410-414; 429; 440-449 (Cardio-, cerebro- and peripheral vacsular disease)	>64 years	β=0.00068 (0.000214)
	Peng et al. (2008)[10]—ICD 426-427; 428; 430-438; 410-414; 429; 440-449 (Cardio-, cerebro- and peripheral vacsular disease)	>64 years	β=0.00071 (0.00013)
	Bell et al. (2008)[11]—ICD 426-427; 428; 430-438; 410-414; 429; 440-449 (Cardio-, cerebro- and peripheral vacsular disease)	>64 years	
	Moolgavkar (2000)[6]—ICD 390–429 (all cardiovascular)	20–64 years	RR=1.04 (t statistic: 4.1) per 10 μg/m^3
Asthma-related ER visits	Mar et al. (2004)[12]	All Ages	RR = 1.04 (1.01 – 1.07) per 7 μg/m^3
	Slaughter et al. (2003)[13]		RR = 1.03 (0.98 – 1.09) per 10 μg/m^3
**Other Health Endpoints**			
Acute bronchitis	Dockery et al. (1996)[14]	8–12 years	OR = 1.50 (0.91 – 2.47) per 14.9 μg/m^3

Upper respiratory symptoms	Pope et al. (1991)[15]	Asthmatics, 9–11 years	1.003 (1—1.006) per 10 μg/m^3
Lower respiratory symptoms	Schwartz and Neas (2000)[16]	7–14 years	OR = 1.11 (1.58 – 1.58) per 15 μg/m^3
Asthma exacerbations	Ostro et al. (2001)[17] (cough, wheeze and shortness of breath)	6–18 years	RR = 1.04 (1.01 – 1.07) per 7 μg/m^3
	Mar et al. (2004) (cough, shortness of breath) [12]		RR = 1.03 (0.98 – 1.09) per 10 μg/m^3
Work loss days	Ostro (1987)[18]	18–65 years	β=0.0046 (0.00036)
Minor Restricted Activity Days (MRADs)	Ostro and Rothschild (1989)[20]	18–65 years	β=0.00220 (0.000658)

[A] Where available, relative risk (RR) and odds ratios (OR) are reported from each epidemiological study. Otherwise, beta coefficients and standard errors are reported from each study. Beta coefficients were derived from each RR or OR using the following equation: (LN(RR or OR))/unit change in pollution.

Table S-2: Epidemiological Study Parameters Used to Quantify Ozone-Attributable Risks

Endpoint	Study	Study Population	Risk Estimate (95th Percentile Confidence Interval)^{A}
**Premature Mortality**			
Time Series	Bell et al. (2004)[21]	All Ages	β = 0.000261 (0.000089)
	Levy et al. (2005)[22]	All Ages	β = 0.001119 (0.000179)
**Hospital & Emergency Department Admissions**			
Respiratory	Katsouyanni et al. (2009)[23]	>65	β = 0.00064 (0.00040)
	Glad et al. (2012)[24]	All Ages	β = 0.00306 (0.00117)
	Ito et al. (2007)[25]	>64 years	β = 0.00521 (0.00091)
	Mar and Koenig (2010)[26]	All ages	β = 0.01044 (0.00436) (0-17 yr olds)<br>β = 0.00770 (0.00284) (18-99 yr olds)
Asthma ED visits	Peel et al. (2005)[27]	All ages	β = 0.00087 (0.00053)
	Sarnat et al. (2013)[28]	All ages	β = 0.00111 (0.00028)
	Wilson et al. (2005)[29]	All ages	RR = 1.022 (0.996 – 1.049)
Asthma Exacerbation	Mortimer et al. (2002)[30]	6-18	β = 0.00929 (0.00387)
	Schildcrout et al. (2006)[31]	6-18	β = 0.00222 (0.00282)
School loss days	Chen et al. (2000)[32]	5-17	β = 0.015763 (0.004985)
	Gilliland et al. (2001)[33]	5-17	β = 0.007824 (0.004445)
Acute Respiratory Symptoms	Ostro and Rothschild[20]	18-65	β = 0.002596 (0.000776)

[A] Where available, relative risk (RR) and odds ratios (OR) are reported from each epidemiological study. Otherwise, beta coefficients and standard errors are reported from each study. Beta coefficients were derived from each RR or OR using the following equation: (LN(RR or OR))/unit change in pollution.

Procedure for projecting death rates to future years

The BenMAP-CE program contains age- and cause-stratified death rates for each county in the contiguous U.S. through the year 2060 in 5-year increments. To estimate these rates, we calculated annual adjustment factors, based on a series of Census Bureau projected national mortality rates (for all-cause mortality), to adjust the age- and county-specific mortality rates calculated using an average of 2012-2014 data from the CDC-WONDER database as a baseline (Table S-3). We used the following procedure:

1. For each age group, we obtained the series of projected national mortality rates from 2013 to 2050 (see the 2013 rate in Table S-3 below) based on Census Bureau projected life tables.

2. We then calculated, separately for each age group, the ratio of Census Bureau national mortality rate in year Y (Y = 2014, 2015, ..., 2060) to the 2013 rate. These ratios are shown for selected years in Table S-4.

3. Finally, to estimate mortality rates in year Y (Y = 2015, 2020, ..., 2060) that are both age-group-specific and county-specific, we multiplied the county- and age-group-specific mortality rates for 2012-2014 by the appropriate ratio calculated in the previous step. For example, to estimate the projected mortality rate in 2015 among ages 18-24 in Wayne County, MI, we multiplied the mortality rate for ages 18-24 in Wayne County in 2012-2014 by the ratio of Census Bureau projected national mortality rate in 2015 for ages 18-24 to Census Bureau national mortality rate in 2013 for ages 18-24.

Table S-3: All-Cause Mortality Rate (per 100 people per year) by Source, Year and Age Group

Source & Year	*18-24*	*25-34*	*35-44*	*45-54*	*55-64*	*65-74*	*75-84*	*85+*
Calculated CDC 2012-2014	0.078	0.107	0.173	0.405	0.862	1.797	4.628	13.580
Census Bureau 2013[A]	0.088	0.102	0.183	0.387	0.930	2.292	5.409	13.091

[A] For a detailed description of the model, the assumptions, and the data used to create Census Bureau projections, see the working paper, “Methodology and Assumptions for the 2012 National Projections,” which is available on http://www.census.gov/population/projections/files/methodology/methodstatement12.pdf

Table S-4: Ratio of Future Year All-Cause Mortality Rate to 2013 Estimated All-Cause Mortality Rate by Age Group

Year	*18-24*	*25-34*	*35-44*	*45-54*	*55-64*	*65-74*	*75-84*	*85+*
2015	0.96	1.02	0.96	0.96	1.01	1.02	1.03	1.00
2020	0.98	1.04	0.97	0.98	1.02	1.03	1.03	1.00
2025	0.74	0.80	0.75	0.77	0.85	0.91	0.93	0.97

Table S-5: Baseline and Prevalence Rates for Included Morbidity Endpoints

Endpoint	*Parameter*	*Rates*	
		Value	*Source*[a]
Hospitalizations	Daily hospitalization rate	Age-, region-, and cause-specific rate	Agency for Healthcare Research and Quality (2014)[35]
Asthma ER Visits	Daily asthma ER visit rate	Age- and region-specific visit rate	Agency for Healthcare Research and Quality (2007) [35]
Nonfatal Myocardial Infarction (heart attacks)	Daily nonfatal myocardial infarction incidence rate per person, 18+	Age-, region-, state-, and county-specific rate	2007 AHRQ data files; adjusted by 0.93 for probability of surviving after 28 days[36]
Asthma Exacerbations	Incidence<br>• daily wheeze<br>• daily cough<br>• daily dyspnea	 0.173<br>0.145<br>0.074	Ostro et al. (2001)[17]
	Prevalence among asthmatic children	0.0780	American Lung Association[37]
Acute Bronchitis	Annual bronchitis incidence rate, children	0.043	American Lung Association (2002) Table 11[37]
Lower Respiratory Symptoms	Daily lower respiratory symptom incidence among children[d]	0.0012	Schwartz and Neas (2000)[16]
Upper Respiratory Symptoms	Daily upper respiratory symptom incidence among asthmatic children	0.3419	Pope et al. (1991, Table 2)[15]
Work Loss Days	Daily WLD incidence rate per person (18–65)<br>• Aged 18–24<br>• Aged 25–44<br>• Aged 45–64	 <br>0.00540<br>0.00678<br>0.00492	US Bureau of the Census (2001)[40]
Minor Restricted-Activity Days	Daily MRAD incidence rate per person	0.02137	Ostro and Rothschild (1989)[20]

[a] Healthcare Cost and Utilization Program (HCUP) database contains individual level, state and regional-level hospital and emergency department discharges for a variety of ICD codes.

[b] See ftp://ftp.cdc.gov/pub/Health_Statistics/NCHS/Datasets/NHDS/.

[c] See ftp://ftp.cdc.gov/pub/Health_Statistics/NCHS/Datasets/NHAMCS/.

[d] Lower respiratory symptoms are defined as two or more of the following: cough, chest pain, phlegm, and wheeze.

Table S-6 $PM_{2.5}$ + Ozone Related Mortality and Morbidity Outcomes Attributable to 17 Mobile Source Sectors in 2011

	Nonroad recreational (incl. pleasure craft)			Nonroad construction			Nonroad lawn & garden commercial			Nonroad lawn & garden residential		
Premature Death	Point Estimate	2.5%	95%	Point Estimate	2.5%	95%	Point Estimate	2.5%	95%	Point Estimate	2.5%	95%
Krewski et al. (2009) & Bell et al. (2004)	720	420	1,000	1,900	1,200	2,600	740	480	990	210	130	290
Lepeule et al. (2012) & Levy et al. (2005)	2,100	1,200	3,000	4,800	2,500	7,000	1,800	950	2,700	560	310	810
Non-Fatal heart attacks												
Peters et al. (2001)	490	120	860	1,600	390	2,800	620	150	1,100	170	40	290
Pooled Estimate of 4 Studies	53	19	140	170	64	470	67	25	180	18	7	48
Cardiovascular Hospital Admissions	120	55	220	410	190	740	160	71	280	43	19	76
Respiratory Hospital Admissions	400	-100	850	630	-150	1,300	230	-60	460	90	-20	180
Respiratory Emergency Department Visits	1,900	44	5,200	2,600	-310	6,500	920	-110	2,300	400	-18	1,100
Acute Bronchitis	790	-180	1,700	2,800	-650	6,100	1,000	-240	2,300	270	-64	600
Acute Respiratory Symptoms	1,700,000	860,000	2,500,000	2,600,000	1,700,000	3,400,000	930,000	610,000	1,300,000	360,000	210,000	510,000
Aggravated Asthma	550,000	-460,000	1,300,000	520,000	-400,000	1,300,000	180,000	-140,000	440,000	98,000	-80,000	240,000
Upper Respiratory Symptoms	14,000	2,600	26,000	50,000	9,100	91,000	19,000	3,400	34,000	4,900	900	9,000
Lower Respiratory Symptoms	10,000	3,800	16,000	35,000	13,000	57,000	13,000	5,000	21,000	3,500	1,300	5,600
Lost Work Days	71,000	60,000	82,000	250,000	210,000	290,000	95,000	81,000	110,000	25,000	21,000	29,000
Lost School Days	400,000	140,000	880,000	340,000	120,000	760,000	120,000	42,000	260,000	68,000	24,000	150,000

	Nonroad agriculture			Nonroad commercial			Nonroad all other: (industrial, logging, mining, oil field)			Onroad light duty gas cars and motorcycles		
Premature Death	Point Estimate	2.5%	95%	Point Estimate	2.5%	95%	Point Estimate	2.5%	95%	Point Estimate	2.5%	95%
Krewski et al. (2009) & Bell et al. (2004)	660	380	940	530	340	720	590	370	800	2,700	1,700	3,700
Lepeule et al. (2012) & Levy et al. (2005)	1,900	1,100	2,800	1,300	700	1,900	1,500	810	2,200	7,100	3,900	10,300
Non-Fatal heart attacks												
Peters et al. (2001)	450	110	780	440	110	770	480	120	830	2,100	520	3,700
Pooled Estimate of 4 Studies	48	18	130	48	18	130	51	19	140	230	84	610
Cardiovascular Hospital Admissions	110	48	190	120	53	210	120	55	220	540	240	970
Respiratory Hospital Admissions	390	-100	830	180	-40	350	220	-60	460	1,100	-280	2,300
Respiratory Emergency Department Visits	1,700	44	4,600	760	-95	1,900	940	-66	2,400	4,800	-210	12,600
Acute Bronchitis	710	-170	1,600	760	-180	1,700	820	-190	1,800	3,600	-850	8,000
Acute Respiratory Symptoms	1,500,000	780,000	2,300,000	720,000	470,000	980,000	900,000	550,000	1,300,000	4,500,000	2,600,000	6,400,000
Aggravated Asthma	540,000	-450,000	1,300,000	150,000	-110,000	350,000	220,000	-180,000	540,000	1,200,000	-980,000	2,900,000
Upper Respiratory Symptoms	13,000	2,400	24,000	14,000	2,500	25,000	15,000	2,700	27,000	66,000	12,000	120,000
Lower Respiratory Symptoms	9,100	3,400	15,000	9,700	3,700	16,000	10,000	4,000	17,000	46,000	17,000	74,000
Lost Work Days	61,000	51,000	70,000	72,000	61,000	83,000	73,000	62,000	85,000	330,000	280,000	380,000
Lost School Days	390,000	140,000	860,000	96,000	34,000	210,000	150,000	54,000	340,000	840,000	300,000	1,900,000

Table S-6 (Continued) PM2.5 + Ozone Related Mortality and Morbidity Outcomes Attributable to 17 Mobile Source Sectors in 2011

	Onroad light duty gas trucks			Onroad heavy duty gas + CNG			Onroad light duty diesel			Onroad heavy duty diesel		
Premature Death	Point Estimate	2.5%	95%	Point Estimate	2.5%	95%	Point Estimate	2.5%	95%	Point Estimate	2.5%	95%
Krewski et al. (2009) & Bell et al. (2004)	3,200	1,900	4,400	130	80	180	320	200	440	5,300	3,300	7,200
Lepeule et al. (2012) & Levy et al. (2005)	8,800	5,000	12,600	360	210	520	840	460	1,200	13,700	7,500	19,900
Non-Fatal heart attacks												
Peters et al. (2001)	2,300	560	4,000	88	21	150	250	62	440	4,200	1,000	7,400
Pooled Estimate of 4 Studies	250	91	660	9	4	25	27	10	73	460	170	1,200
Cardiovascular Hospital Admissions	580	260	1,000	22	10	40	65	29	120	1,100	480	1,900
Respiratory Hospital Admissions	1,600	-390	3,300	70	-20	140	130	-30	270	2,100	-540	4,400
Respiratory Emergency Department Visits	7,200	-24	22,000	320	3	890	550	-26	1,500	8,700	-450	25,000
Acute Bronchitis	3,900	-920	8,700	150	-34	330	450	-100	1,000	7,100	-1,700	16,000
Acute Respiratory Symptoms	6,500,000	3,500,000	9,400,000	280,000	150,000	410,000	540,000	320,000	760,000	8,400,000	5,000,000	11,800,000
Aggravated Asthma	2,000,000	-1,700,000	4,900,000	92,000	-76,000	220,000	140,000	-120,000	350,000	2,200,000	-1,800,000	5,400,000
Upper Respiratory Symptoms	71,000	13,000	130,000	2,700	480	4,800	8,100	1,500	15,000	130,000	24,000	240,000
Lower Respiratory Symptoms	50,000	19,000	81,000	1,900	710	3,000	5,700	2,200	9,200	90,000	34,000	146,000
Lost Work Days	350,000	300,000	400,000	13,000	11,000	15,000	40,000	34,000	46,000	630,000	530,000	730,000
Lost School Days	1,400,000	510,000	3,200,000	65,000	23,000	150,000	99,000	35,000	220,000	1,500,000	550,000	3,400,000

	c1c2 marine			c3 marine			Rail			Aircraft (excluding ground support)			Aircraft ground support only		
Premature Death	Point Estimate	2.5%	95%	Point Estimate	2.5%	95%	Point Estimate	2.5%	95%	Point Estimate	2.5%	95%	Point Estimate	2.5%	95%
Krewski et al. (2009) & Bell et al. (2004)	510	300	720	1,900	1,200	2,600	1,100	680	1,500	380	250	520	26	16	35
Lepeule et al. (2012) & Levy et al. (2005)	1,500	840	2,100	4,900	2,600	7,100	3,000	1,700	4,300	930	490	1,400	65	35	95
Non-Fatal heart attacks															
Peters et al. (2001)	350	86	610	1,500	370	2,700	850	210	1,500	330	81	580	21	5	37
Pooled Estimate of 4 Studies	38	14	100	160	61	440	92	34	250	36	13	96	2	1	6
Cardiovascular Hospital Admissions	91	41	160	400	180	710	210	94	370	85	38	150	5	2	10
Respiratory Hospital Admissions	280	-70	600	690	-180	1,400	500	-130	1,100	120	-30	230	9	-2	19
Respiratory Emergency Department Visits	1,300	-2	3,500	2,900	-210	7,500	2,000	-39	5,500	450	-64	1,100	40	-3	100
Acute Bronchitis	550	-130	1,200	2,700	-630	5,900	1,400	-330	3,100	580	-140	1,300	36	-8	80
Acute Respiratory Symptoms	1,100,000	580,000	1,600,000	2,900,000	1,800,000	4,100,000	2,000,000	1,100,000	2,800,000	490,000	330,000	650,000	40,000	24,000	56,000
Aggravated Asthma	360,000	-300,000	860,000	690,000	-550,000	1,700,000	580,000	-480,000	1,400,000	90,000	-67,000	220,000	9,900	-7,900	24,000
Upper Respiratory Symptoms	10,000	1,800	18,000	49,000	8,800	88,000	26,000	4,600	46,000	11,000	1,900	19,000	650	120	1,200
Lower Respiratory Symptoms	7,000	2,600	11,000	34,000	13,000	55,000	18,000	6,800	29,000	7,400	2,800	12,000	460	170	740
Lost Work Days	54,000	46,000	62,000	250,000	210,000	280,000	120,000	100,000	140,000	53,000	45,000	61,000	3,300	2,800	3,800
Lost School Days	250,000	90,000	560,000	470,000	170,000	1,000,000	410,000	140,000	910,000	57,000	20,000	130,000	6,700	2,400	15,000

Table S-7 PM2.5 + Ozone Related Mortality and Morbidity Outcomes Attributable to 17 Mobile Source Sectors in 2025

	Nonroad recreational (incl. pleasure craft)			Nonroad construction			Nonroad lawn & garden commercial			Nonroad lawn & garden residential		
Premature Death	Point Estimate	2.5%	95%	Point Estimate	2.5%	95%	Point Estimate	2.5%	95%	Point Estimate	2.5%	95%
Krewski et al. (2009) & Bell et al. (2004)	520	280	760	840	520	1,200	1,100	710	1,500	300	190	410
Lepeule et al. (2012) & Levy et al. (2005)	1,700	1,000	2,400	2,300	1,200	3,300	2,700	1,400	3,900	760	410	1,100
Non-Fatal heart attacks												
Peters et al. (2001)	310	76	550	710	170	1,200	1,000	250	1,800	280	67	480
Pooled Estimate of 4 Studies	34	12	90	80	28	210	110	41	290	30	11	79
Cardiovascular Hospital Admissions	78	34	140	180	81	340	260	110	480	71	31	130
Respiratory Hospital Admissions	470	-120	1,000	430	-120	910	390	-120	800	120	-36	250
Respiratory Emergency Department Visits	1,700	91	4,800	1,500	-61	4,000	1,200	-160	3,100	420	-39	1,100
Acute Bronchitis	400	-93	880	1,000	-240	2,300	1,400	-320	3,100	380	-89	840
Acute Respiratory Symptoms	1,400,000	650,000	2,100,000	1,300,000	770,000	1,900,000	1,200,000	810,000	1,600,000	380,000	240,000	520,000
Aggravated Asthma	510,000	-430,000	1,200,000	370,000	-300,000	890,000	230,000	-170,000	540,000	85,000	-66,000	200,000
Upper Respiratory Symptoms	7,100	1,300	13,000	18,000	3,300	33,000	25,000	4,500	45,000	6,800	1,200	12,000
Lower Respiratory Symptoms	5,000	1,900	8,100	13,000	4,900	21,000	18,000	6,700	28,000	4,800	1,800	7,800
Lost Work Days	36,000	30,000	41,000	93,000	79,000	110,000	130,000	110,000	150,000	35,000	29,000	40,000
Lost School Days	370,000	130,000	820,000	260,000	90,000	570,000	150,000	51,000	320,000	57,000	20,000	130,000

	Nonroad agriculture			Nonroad commercial			Nonroad all other: (industrial, logging, mining, oil field)			Onroad light duty gas cars and motorcycles		
Premature Death	Point Estimate	2.5%	95%	Point Estimate	2.5%	95%	Point Estimate	2.5%	95%	Point Estimate	2.5%	95%
Krewski et al. (2009) & Bell et al. (2004)	340	180	500	450	290	620	340	210	480	1,700	1,100	2,300
Lepeule et al. (2012) & Levy et al. (2005)	1,100	690	1,600	1,200	640	1,700	960	540	1,400	4,300	2,300	6,200
Non-Fatal heart attacks												
Peters et al. (2001)	200	50	360	410	99	710	280	69	490	1,600	380	2,700
Pooled Estimate of 4 Studies	22	8	59	44	16	120	30	11	81	170	62	450
Cardiovascular Hospital Admissions	49	22	91	110	47	200	71	31	130	400	180	740
Respiratory Hospital Admissions	300	-78	660	210	-59	430	200	-56	430	710	-220	1,500
Respiratory Emergency Department Visits	1,000	51	2,800	760	-57	2,000	710	-8	1,900	2,200	-200	5,700
Acute Bronchitis	270	-63	600	580	-130	1,300	390	-92	880	2,200	-520	4,900
Acute Respiratory Symptoms	850,000	410,000	1,300,000	660,000	400,000	910,000	610,000	340,000	890,000	2,200,000	1,400,000	3,000,000
Aggravated Asthma	330,000	-280,000	800,000	160,000	-120,000	380,000	190,000	-150,000	450,000	490,000	-380,000	1,200,000
Upper Respiratory Symptoms	4,800	880	8,800	10,000	1,900	19,000	7,100	1,300	13,000	40,000	7,200	72,000
Lower Respiratory Symptoms	3,400	1,300	5,500	7,300	2,800	12,000	5,000	1,900	8,100	28,000	11,000	45,000
Lost Work Days	23,000	19,000	26,000	54,000	46,000	63,000	36,000	30,000	41,000	200,000	170,000	230,000
Lost School Days	240,000	84,000	530,000	107,000	38,000	240,000	130,000	46,000	290,000	320,000	110,000	720,000

Table S-7 (Continued) PM2.5 + Ozone Related Mortality and Morbidity Outcomes Attributable to 17 Mobile Source Sectors in 2025

	Onroad light duty gas trucks			Onroad heavy duty gas + CNG			Onroad light duty diesel			Onroad heavy duty diesel		
Premature Death	Point Estimate	2.5%	95%	Point Estimate	2.5%	95%	Point Estimate	2.5%	95%	Point Estimate	2.5%	95%
Krewski et al. (2009) & Bell et al. (2004)	1,800	1,100	2,500	100	61	140	610	360	850	2,500	1,500	3,400
Lepeule et al. (2012) & Levy et al. (2005)	4,800	2,600	7,000	290	160	410	1,700	970	2,500	7,000	4,000	10,000
Non-Fatal heart attacks												
Peters et al. (2001)	1,600	400	2,800	80	20	140	490	120	860	2,000	480	3,400
Pooled Estimate of 4 Studies	180	65	470	9	3	23	53	20	140	210	78	570
Cardiovascular Hospital Admissions	420	180	760	21	9	38	130	55	230	500	220	910
Respiratory Hospital Admissions	890	-260	1,900	62	-17	130	370	-100	790	1,500	-420	3,200
Respiratory Emergency Department Visits	2,900	-150	7,600	220	-1	590	1,300	-6	3,500	5,100	-19	15,000
Acute Bronchitis	2,300	-540	5,100	110	-26	250	690	-160	1,500	2,700	-630	6,000
Acute Respiratory Symptoms	2,800,000	1,600,000	3,900,000	190,000	100,000	270,000	1,100,000	620,000	1,700,000	4,400,000	2,400,000	6,500,000
Aggravated Asthma	710,000	-570,000	1,700,000	58,000	-48,000	140,000	350,000	-290,000	840,000	1,400,000	-1,100,000	3,300,000
Upper Respiratory Symptoms	42,000	7,600	76,000	2,000	360	3,600	13,000	2,300	23,000	49,000	8,800	88,000
Lower Respiratory Symptoms	29,000	11,000	47,000	1,400	540	2,300	8,800	3,300	14,000	34,000	13,000	55,000
Lost Work Days	210,000	180,000	240,000	10,000	8,500	12,000	63,000	54,000	73,000	240,000	200,000	280,000
Lost School Days	490,000	170,000	1,100,000	41,000	15,000	92,000	250,000	87,000	550,000	980,000	350,000	2,200,000

	c1c2 marine			c3 marine			Rail			Aircraft (excluding ground support)			Aircraft ground support only		
Premature Death	Point Estimate	2.5%	95%	Point Estimate	2.5%	95%	Point Estimate	2.5%	95%	Point Estimate	2.5%	95%	Point Estimate	2.5%	95%
Krewski et al. (2009) & Bell et al. (2004)	390	220	560	800	400	1,100	1,000	590	1,400	650	410	880	42	25	58
Lepeule et al. (2012) & Levy et al. (2005)	1,200	730	1,700	2,500	1,600	3,500	3,000	1,700	4,200	1,700	900	2,400	120	65	170
Non-Fatal heart attacks															
Peters et al. (2001)	270	66	470	470	110	820	750	180	1,300	590	140	1,000	35	9	61
Pooled Estimate of 4 Studies	29	11	80	50	19	140	81	30	220	63	23	170	4	1	10
Cardiovascular Hospital Admissions	70	31	130	120	54	230	180	81	340	150	67	280	9	4	17
Respiratory Hospital Admissions	320	-83	700	720	-190	1,600	680	-180	1,500	290	-86	610	24	-7	51
Respiratory Emergency Department Visits	1,100	28	3,100	2,800	140	8,700	2,200	36	6,100	970	-70	2,500	88	-2	240
Acute Bronchitis	360	-84	800	650	-150	1,500	1,000	-240	2,300	830	-190	1,800	49	-12	110
Acute Respiratory Symptoms	910,000	460,000	1,400,000	2,300,000	1,100,000	3,400,000	1,900,000	1,000,000	2,900,000	930,000	560,000	1,300,000	78,000	43,000	110,000
Aggravated Asthma	320,000	-270,000	760,000	810,000	-690,000	2,000,000	650,000	-540,000	1,600,000	220,000	-180,000	530,000	23,000	-19,000	56,000
Upper Respiratory Symptoms	6,500	1,200	12,000	12,000	2,100	21,000	18,000	3,300	33,000	15,000	2,700	27,000	890	160	1,600
Lower Respiratory Symptoms	4,600	1,700	7,400	8,300	3,200	13,000	13,000	4,900	21,000	11,000	4,000	17,000	630	240	1,000
Lost Work Days	35,000	30,000	40,000	62,000	52,000	71,000	89,000	75,000	100,000	77,000	65,000	89,000	4,600	3,900	5,300
Lost School Days	230,000	80,000	500,000	590,000	210,000	1,300,000	470,000	170,000	1,000,000	150,000	53,000	330,000	16,000	5,700	36,000

References

(1) Krewski, D.; Jerrett, M.; Burnett, R. T.; Ma, R.; Hughes, E.; Shi, Y.; Turner, M. C.; Pope, C. A.; Thurston, G.; Calle, E. E.; et al. Extended Follow-up and Spatial Analysis of the American Cancer Society Study Linking Particulate Air Pollution and Mortality. *Res. Rep. Health. Eff. Inst.* **2009**, No. 140, 5–114; discussion 115-36.

(2) Lepeule, J.; Laden, F.; Dockery, D. W.; Schwartz, J. D. Chronic Exposure to Fine Particles and Mortality: An Extended Follow-up of the Harvard Six Cities Study from 1974 to 2009. National Institute of Environmental Health Sciences 2012.

(3) Peters, A.; Dockery, D. W.; Muller, J. E.; Mittleman, M. A. Increased Particulate Air Pollution and the Triggering of Myocardial Infarction. *Circulation* **2001**, *103* (23), 2810–2815. https://doi.org/10.1161/01.CIR.103.23.2810.

(4) Babin, S. M.; Burkom, H. S.; Holtry, R. S.; Tabernero, N. R.; Stokes, L. D.; Davies-Cole, J. O.; DeHaan, K.; Lee, D. H. Pediatric Patient Asthma-Related Emergency Department Visits and Admissions in Washington, DC, from 2001-2004, and Associations with Air Quality, Socio-Economic Status and Age Group. *Environ. Health* **2007**, *6*, 9. https://doi.org/10.1186/1476-069X-6-9.

(5) Moolgavkar, S. H. Air Pollution and Hospital Admissions for Diseases of the Circulatory System in Three U.S. Metropolitan Areas. *J. Air Waste Manag. Assoc.* **2000**, *50* (7), 1199–1206.

(6) Moolgavkar, S. H. Air Pollution and Hospital Admissions for Diseases of the Circulatory System in Three U.S. Metropolitan Areas. *J. Air Waste Manage. Assoc.* **2000**, *50* (7), 1199–1206. https://doi.org/10.1080/10473289.2000.10464162.

(7) Zanobetti, A.; Schwartz, J. Air Pollution and Emergency Admissions in Boston, MA. *J. Epidemiol. Community Health* **2006**, *60* (10), 890–895. https://doi.org/10.1136/jech.2005.039834.

(8) Zanobetti, A.; Schwartz, J. The Effect of Fine and Coarse Particulate Air Pollution on Mortality: A National Analysis. *Environ. Health Perspect.* **2009**, *117* (6), 898–903. https://doi.org/10.1289/ehp.0800108.

(9) Peng, R. D.; Bell, M. L.; Geyh, A. S.; McDermott, A.; Zeger, S. L.; Samet, J. M.; Dominici, F. Emergency Admissions for Cardiovascular and Respiratory Diseases and the Chemical Composition of Fine Particle Air Pollution. *Environ. Health Perspect.* **2009**, *117* (6), 957–963. https://doi.org/10.1289/ehp.0800185.

(10) Peng, R. D.; Chang, H. H.; Bell, M. L.; McDermott, A.; Zeger, S. L.; Samet, J. M.; Dominici, F. Coarse Particulate Matter Air Pollution and Hospital Admissions for Cardiovascular and Respiratory Diseases among Medicare Patients. *JAMA* **2008**, *299* (18), 2172–2179. https://doi.org/10.1001/jama.299.18.2172.

(11) Bell, M. L.; Ebisu, K.; Peng, R. D.; Walker, J.; Samet, J. M.; Zeger, S. L.; Dominici, F. Seasonal and Regional Short-Term Effects of Fine Particles on Hospital Admissions in 202 US Counties, 1999-2005. *Am. J. Epidemiol.* **2008**, *168* (11), 1301–1310. https://doi.org/10.1093/aje/kwn252.

(12) Mar, T. F.; Larson, T. V; Stier, R. a; Claiborn, C.; Koenig, J. Q. An Analysis of the Association between Respiratory Symptoms in Subjects with Asthma and Daily Air Pollution in Spokane,

Washington. *Inhal. Toxicol.* **2004**, *16* (13), 809–815. https://doi.org/10.1080/08958370490506646.

(13) Slaughter, J. C.; Lumley, T.; Sheppard, L.; Koenig, J. Q.; Shapiro, G. G. Effects of Ambient Air Pollution on Symptom Severity and Medication Use in Children with Asthma. *Ann. Allergy. Asthma Immunol.* **2003**, *91* (4), 346–353. https://doi.org/10.1016/S1081-1206(10)61681-X.

(14) Dockery, D. W.; Cunningham, J.; Damokosh, a I.; Neas, L. M.; Spengler, J. D.; Koutrakis, P.; Ware, J. H.; Raizenne, M.; Speizer, F. E. Health Effects of Acid Aerosols on North American Children: Respiratory Symptoms. *Environ. Health Perspect.* **1996**, *104* (5), 500–505.

(15) Pope, C. A.; Dockery, D. W.; Spengler, J. D.; Raizenne, M. E. Respiratory Health and PM10 Pollution. A Daily Time Series Analysis. *Am. Rev. Respir. Dis.* **1991**, *144* (3 Pt 1), 668–674.

(16) Schwartz, J.; Neas, L. M. Fine Particles Are More Strongly Associated than Coarse Particles with Acute Respiratory Health Effects in Schoolchildren. *Epidemiology* **2000**, *11* (1), 6–10.

(17) Ostro, B.; Lipsett, M.; Mann, J.; Braxton-Owens, H.; White, M. Air Pollution and Exacerbation of Asthma in African-American Children in Los Angeles. *Epidemiology* **2001**, *12* (2), 200–208.

(18) Ostro, B. D. Air Pollution and Morbidity Revisited: A Specification Test. *J. Environ. Econ. Manage.* **1987**, *14* (1), 87–98. https://doi.org/10.1016/0095-0696(87)90008-8.

(19) Ostro, B. D.; Rothschild, S. Air Pollution and Acute Respiratory Morbidity: An Observational Study of Multiple Pollutants. *Environ. Res.* **1989**, *50* (2), 238–247. https://doi.org/10.1016/S0013-9351(89)80004-0.

(20) Ostro, B. D.; Rothschild, S. Air Pollution and Acute Respiratory Morbidity: An Observational Study of Multiple Pollutants. *Environ. Res.* **1989**, *50* (2), 238–247. https://doi.org/10.1016/S0013-9351(89)80004-0.

(21) Smith, R. L.; Xu, B.; Switzer, P. Reassessing the Relationship between Ozone and Short-Term Mortality in U.S. Urban Communities. *Inhal. Toxicol.* **2009**, *21 Suppl* 2 (June), 37–61. https://doi.org/10.1080/08958370903161612.

(22) Zanobetti, A.; Schwartz, J. Is There Adaptation in the Ozone Mortality Relationship: A Multi-City Case-Crossover Analysis. *Environ. Health* **2008**, 7, 22. https://doi.org/10.1186/1476-069X-7-22.

(23) Katsouyanni, K.; Samet, J. M.; Anderson, H. R.; Atkinson, R.; Le Tertre, A.; Medina, S.; Samoli, E.; Touloumi, G.; Burnett, R. T.; Krewski, D.; et al. Air Pollution and Health: A European and North American Approach (APHENA). *Res. Rep. Heal. Eff. Inst.* **2009**, No. 142, 5–90.

(24) Glad, J. A.; Brink, L. L.; Talbott, E. O.; Lee, P. C.; Xu, X.; Saul, M.; Rager, J. The Relationship of Ambient Ozone and PM2.5 Levels and Asthma Emergency Department Visits: Possible Influence of Gender and Ethnicity. *Archives of Environmental and Occupational Health*. 2012, pp 103–108. https://doi.org/10.1080/19338244.2011.598888.

(25) Ito, K.; Thurston, G. D.; Silverman, R. a. Characterization of PM2.5, Gaseous Pollutants, and Meteorological Interactions in the Context of Time-Series Health Effects Models. *J. Expo. Sci. Environ. Epidemiol.* **2007**, *17 Suppl* 2, S45-60. https://doi.org/10.1038/sj.jes.7500627.

(26) Mar, T. F.; Koenig, J. Q. Relationship between Visits to Emergency Departments for Asthma and Ozone Exposure in Greater Seattle, Washington . *Annals of Allergy, Asthma, and Immunology*. 2009, pp 474–479. https://doi.org/10.1016/S1081-1206(10)60263-3.

(27) Peel, J. L.; Tolbert, P. E.; Klein, M.; Metzger, K. B.; Flanders, W. D.; Todd, K.; Mulholland, J. A.; Ryan, P. B.; Frumkin, H. Ambient Air Pollution and Respiratory Emergency Department Visits.

Epidemiology **2005**, *16* (2), 164–174.

(28) Sarnat, J. A.; Sarnat, S. E.; Flanders, W. D.; Chang, H. H.; Mulholland, J.; Baxter, L.; Isakov, V.; Ozkaynak, H. Spatiotemporally Resolved Air Exchange Rate as a Modifier of Acute Air Pollution-Related Morbidity in Atlanta. *Journal of Exposure Science and Environmental Epidemiology*. 2013, pp 606–615. https://doi.org/10.1038/jes.2013.32.

(29) Wilson, A. M.; Wake, C. P.; Kelly, T.; Salloway, J. C. Air Pollution, Weather, and Respiratory Emergency Room Visits in Two Northern New England Cities: An Ecological Time-Series Study. *Environ. Res.* **2005**, *97* (3), 312–321. https://doi.org/10.1016/j.envres.2004.07.010.

(30) Mortimer, K. M.; Neas, L. M.; Dockery, D. W.; Redline, S.; Tager, I. B. The Effect of Air Pollution on Inner-City Children with Asthma. *Eur. Respir. J.* **2002**, *19* (4), 699–705. https://doi.org/10.1183/09031936.02.00247102.

(31) Schildcrout, J. S.; Sheppard, L.; Lumley, T.; Slaughter, J. C.; Koenig, J. Q.; Shapiro, G. G. Ambient Air Pollution and Asthma Exacerbations in Children: An Eight-City Analysis. *Am. J. Epidemiol.* **2006**, *164* (6), 505–517. https://doi.org/10.1093/aje/kwj225.

(32) Chen, Lei Jennison, Brian L. Yang, Wei Omaye, S. T. Elementary School Absenteeism and Air Pollution. *Inhal. Toxicol.* **2000**, *12* (11), 20. https://doi.org/http://dx.doi.org/10.1080/08958370050164626.

(33) Gilliland, F. D.; Berhane, K.; Rappaport, E. B.; Thomas, D. C.; Avol, E.; Gauderman, W. J.; London, S. J.; Margolis, H. G.; McConnell, R.; Islam, K. T.; et al. The Effects of Ambient Air Pollution on School Absenteeism Due to Respiratory Illnesses. *Epidemiology* **2001**, *12* (1), 43–54.

(34) Agency for Healthcare Research and Quality. *HCUPNet*; Rockville, MD, 2014.

(35) Agency for Healthcare Research and Quality. *Healthcare Cost and Utilization Project (HCUP)*; Rockville, MD, 2014.

(36) Rosamond, W. Comparison of Medical Care and Survival of Hospitalized Patients with Acute Myocardial Infarction in Poland and the United States. *Am. J. Cardiol.* **1999**, *83* (8), 1180–1185. https://doi.org/10.1016/S0002-9149(99)00056-9.

(37) Trends in Asthma Morbidity and Mortality American Lung Association Epidemiology and Statistics Unit Research and Program Services Division February 2010. *Program* **2010**, No. February.

(38) American Lung Association. *Trends in Asthma Morbidity and Mortality*; Washington, DC, 2010.

(39) Schwartz, J. PM10, Ozone, and Hospital Admissions for the Elderly in Minneapolis-St. Paul, Minnesota. *Arch. Environ. Health* **1994**, *49* (5), 366–374. https://doi.org/10.1080/00039896.1994.9954989.

(40) US Bureau of the Census. *Statistical Abstract of the United States, 2001*; Washington, DC, **2001**.
